# Impact of Comorbidities on the Prevalence and Recovery Outcomes in Elderly Patients With Neck of Femur Fractures

**DOI:** 10.7759/cureus.84502

**Published:** 2025-05-20

**Authors:** Zahir Khan, Muhammad Arsalan Azmat Swati, Aimon Zia, Anwar Imran, Ashraf, Rumman, Amjad Ali

**Affiliations:** 1 Orthopaedic Surgery, MTI Mardan Medical Complex and Bacha Khan Medical College, Mardan, PAK; 2 Orthopaedics and Trauma, MTI Mardan Medical Complex, Mardan, PAK; 3 Orthopaedic Surgery, Jinnah Teaching Hospital, Peshawar, PAK; 4 Research and Development, Pro-Gene Diagnostics and Research Laboratory, Mardan, PAK; 5 Pharmacovigilance/Active Drug Safety Monitoring and Management, Mardan Medical Complex Teaching Hospital, Mardan, PAK; 6 Medicine, Bacha Khan Medical College, Mardan, PAK

**Keywords:** comorbidities, elderly, neck of femur fractures, postoperative complications, recovery outcomes, rehabilitation

## Abstract

Background

Neck of femur (NOF) fractures are a major cause of morbidity and mortality in the elderly, with incidence rising due to the aging population. The presence of comorbidities in elderly patients can increase the risk of complications and extend recovery time. Understanding how these health conditions influence recovery is crucial for improving patient outcomes. The high mortality rates and functional decline linked to NOF fractures emphasize the need for tailored treatment strategies.

Objective

To examine how comorbidities affect the prevalence, recovery outcomes, and overall prognosis of neck of femur fractures in elderly trauma patients.

Materials and methods

A retrospective cohort study was conducted at the Orthopedic Department of Mardan Medical Complex, Pakistan, between December 2023 and March 2025. The study focused on elderly patients (aged ≥65 years) diagnosed with neck of femur (NOF) fractures. Data were extracted from patient medical records, including demographic information, comorbidities, medical history, and recovery outcomes. Statistical analyses, such as descriptive statistics, chi-square tests, and correlation analyses, were performed to investigate the relationships between comorbidities and all studied variables. Additionally, data visualizations, including boxplots, barplots, and heatmaps, were utilized to further explore the associations between comorbidities and all studied variables.

Results

The study population consisted of 163 patients, with 70.55% males (115 patients) and 29.45% females (48 patients). The mean age was 80.23 ± 9.02 years. Anemia had the highest prevalence, affecting 71.77% (117 patients), followed by hypertension in 60.12% (98 patients), diabetes in 57.05% (93 patients), and cardiovascular disease in 55.21% (90 patients). Postoperative complications were common, with hyperglycemia (31.90%, 52 patients), deep vein thrombosis (18.40%, 30 patients), and heart failure (12.88%, 21 patients) being the most frequent. Comorbidities such as anemia, diabetes, and cardiovascular diseases were strongly associated with longer hospital stays, delayed surgery, extended rehabilitation periods, and reduced follow-up care compliance. Additionally, a significant correlation was found between the number of comorbidities and higher pain levels, particularly in patients with anemia and diabetes.

Conclusion

Comorbidities notably impair recovery in elderly patients with NOF fractures, with anemia, hypertension, diabetes, and cardiovascular diseases contributing to delayed recovery, complications, and higher healthcare costs. Early intervention, personalized treatment plans, and targeted rehabilitation are crucial to improve outcomes. This study highlights the importance of thorough preoperative assessments and ongoing management of chronic conditions in this patient population.

## Introduction

Neck of femur (NOF) fractures represent a significant health challenge, particularly among the elderly, who often present with multiple comorbidities that complicate recovery outcomes and overall prognosis. As the global population ages, the incidence of such fractures is on the rise, with mortality rates reported to range between 20% to 30% within the year following the injury, highlighting a substantial burden on healthcare systems globally [1,2]. Understanding the interplay between comorbidities and NOF fractures in older adults is crucial for improving postoperative outcomes and implementing tailored care strategies.

Comorbidities such as cardiovascular diseases, diabetes, and cognitive impairments are prevalent in elderly populations and frequently complicate the clinical management of hip fractures. Studies indicate that patients with multiple comorbid conditions face higher rates of surgical complications, prolonged rehabilitation periods, and an overall decline in functional status post-fracture [1,3]. For instance, cardiovascular diseases can influence anesthesia tolerance and increase risks associated with surgical interventions, while diabetes may lead to delayed wound healing and higher infection rates [2,3]. The presence of cognitive impairments, such as dementia, can further hinder rehabilitation efforts, which are critical for recovery [4,5].

Elderly patients with NOF fractures often demonstrate a combined effect of comorbidities that exacerbates functional decline, leading to significant risks of long-term disability and loss of independence [6,7]. Patients with chronic conditions may experience higher risks for non-union and avascular necrosis due to impaired physiological processes [8,9]. Furthermore, the economic ramifications of managing such complex cases are considerable; prolonged hospital stays and extensive rehabilitation efforts contribute to increased healthcare costs, underscoring the need for effective, individualized treatment protocols [10,11].

Despite the acknowledgment of the critical role that comorbidities play in the outcomes of NOF fractures, there remains a gap in the literature regarding their specific impacts across diverse elderly populations [12,13]. Most existing studies have focused on singular comorbid conditions rather than examining the synergistic effects of multiple health issues on recovery trajectories. This lack of comprehensive research inhibits the development of effective clinical guidelines that could improve management strategies for elderly patients suffering from NOF fractures [14,15].

This research aims to evaluate how specific comorbidities impact recovery, complication rates, and long-term health outcomes in elderly patients with neck of femur fractures (NOF). By identifying key factors that contribute to unfavorable recovery, the study seeks to inform clinical practice and promote personalized care approaches that enhance patient outcomes and quality of life. The increasing frequency of NOF fractures and the complex health needs of the aging population highlight the urgency of this investigation and the need for effective clinical interventions. The growing prevalence of the aging population and comorbidities underscores the need for evidence-based strategies to improve outcomes for elderly patients with hip fractures. This study focuses on understanding how comorbid conditions influence treatment, recovery, and long-term health outcomes, offering a deeper insight into the challenges faced by elderly patients with multiple comorbidities. The findings aim to inform healthcare policies to better support this vulnerable population. Given the importance of early intervention and comprehensive care, the study is expected to contribute significantly to both clinical practice and health policy.

## Materials and methods

Study design and setting

This retrospective cohort study was conducted at the Orthopedic Department of Mardan Medical Complex in Khyber Pakhtunkhwa, Pakistan, between December 2023 and March 2025. The primary objective was to assess the impact of comorbidities on the prevalence, recovery outcomes, and overall prognosis in elderly patients with neck of femur fractures. A convenience sampling method was employed, utilizing data extracted from medical records, rehabilitation outcomes, and postoperative care compliance for elderly trauma patients who were admitted and available during the study period. This design enabled the use of existing medical records to gather necessary data, focusing on patients who met the inclusion criteria.

Inclusion and exclusion criteria

The study included elderly patients aged 65 years or older who were diagnosed with a neck of femur fracture. Inclusion criteria included: (1) patients aged 65 or older diagnosed with a neck of femur fracture, (2) patients with documented comorbidities in their medical records, (3) patients who underwent either surgical or non-surgical management for the fracture, and (4) availability of complete medical records from admission to discharge, including rehabilitation and follow-up details. Exclusion criteria comprised: (1) patients with incomplete medical records, particularly those lacking information on comorbidities or outcomes, (2) patients who had undergone prior orthopedic surgeries unrelated to the current fracture, and (3) patients with advanced malignancies or terminal illnesses that could potentially influence recovery or rehabilitation outcomes.

Sample size calculation

The sample size for this study was calculated to evaluate the effect of comorbidities on the prevalence, recovery outcomes, and prognosis of neck of femur fractures in elderly trauma patients. A power analysis was performed to determine the necessary sample size to detect significant differences in clinical outcomes between patients with and without comorbidities. Using a significance level (α) of 0.05 and a desired power of 80% (β = 0.20), the analysis suggested that a minimum of 160 patients would be required to detect significant differences between the two groups [16]. Given the possibility of missing data and incomplete medical records, a conservative 2% increase was applied to the calculated sample size, resulting in a final sample size of 163 patientsto ensure statistical power and robustness in the analysis​​​​​​**.** This sample size provides sufficient statistical power to detect meaningful associations between comorbidities and clinical outcomes, ensuring reliable and valid results.

Data collection

Data collection was performed through a review of hospital medical records, which included demographic data, clinical history, treatment, and rehabilitation outcomes. Information collected included age, gender, body mass index (BMI), smoking status, and physical activity levels (categorized as sedentary, lightly active, moderately active, or very active). Data on comorbidities were also extracted. Details regarding the nature of surgical interventions (e.g., open reduction and internal fixation (ORIF), hemiarthroplasty, total hip replacement) and non-surgical management (e.g., casting, traction, pain management) were recorded. Rehabilitation data, including the type of rehabilitation received (hospital-based or outpatient), as well as recovery outcomes, such as length of hospital stay, time to surgery, duration of physical therapy, follow-up visits, postoperative complications, and follow-up care compliance, were also documented.

Outcome measures

The clinical outcomes assessed in this study included the length of hospital stay, time to surgery, duration of physical therapy, follow-up visits, postoperative complications, and follow-up care compliance.

Statistical analysis

Statistical analyses were performed using R (version 4.4.2; R Foundation for Statistical Computing, Vienna, Austria) for data analysis and visualization. The normality of continuous variables was assessed using the Shapiro-Wilk test. Continuous variables were presented as means with standard deviations (SD), while categorical variables were summarized using frequencies and percentages. Boxplots and correlation heatmaps were used to compare comorbidities with continuous variables such as age, BMI, length of hospital stay, time to surgery (in hours), duration of physical therapy, and follow-up visits. Bar plots and chi-square tests were used to compare comorbidities with categorical variables, including gender, smoking status, physical activity level, surgical history, surgical approach, pain levels (Visual Analog Scale), preoperative functional status (Barthel Index), type of rehabilitation, and follow-up care compliance. A heatmap was used to examine the frequency of postoperative complications in relation to the number of comorbidities. A p-value of <0.05 was considered statistically significant for all analyses.

Ethical considerations

Ethical approval for this study was obtained from the Institutional Review Board (IRB) of Mardan Medical Complex and Bacha Khan Medical College (NO:792/BKMC). Given the retrospective nature of the study, patient consent was not required. Patient data were anonymized to maintain confidentiality, and appropriate data security measures were implemented to safeguard patient privacy. No additional financial or medical burdens were imposed on the participants during the course of the study.

## Results

The study sample included 163 patients, with 70.55% males (115 patients) and 29.45% females (48 patients). The average age was 80.23 ± 9.02 years, and the average BMI was 30.28 ± 8.74 kg/m^2^, indicating that most participants were overweight or obese. About 29.44% (48 patients) were smokers, while 70.55% (115 patients) were non-smokers. In terms of physical activity, 23.92% (39 patients) were lightly active, 30.06% (49 patients) moderately active, 24.53% (40 patients) sedentary, and 21.47% (35 patients) very active (Table 1).

**Table 1:** Demographic and Clinical Characteristics of Patients Data is presented as frequencies and percentages or means and standard deviation.

Characteristics	Values
Total number of patients	163 (100%)
Age (years)	80.23±9.02
Gender	
Male	115 (70.55%)
Female	48 (29.45%)
BMI (kg/m^2^)	30.28±8.74
Smoking Status	
Yes	48 (29.44%)
No	115 (70.55%)
Physical Activity Level	
Lightly Active	39 (23.92%)
Moderately Active	49 (30.06%)
Sedentary	40 (24.53%)
Very Active	35 (21.47%)

The study population had a range of surgical histories, with 9.20% (15 patients) having a history of joint replacements, 9.81% (16 patients) having previous fractures, and 80.98% (132 patients) having no surgical history. Regarding surgical approaches, 20.85% (34 patients) received a non-surgical approach (casting or traction), 21.47% (36 patients) underwent pain management and rehabilitation, 16.56% (27 patients) had hemiarthroplasty, 22.69% (37 patients) underwent open reduction and internal fixation (ORIF), and 17.79% (29 patients) received total hip replacement (THR). The most common comorbidities were anemia (71.77%, 117 patients), hypertension (60.12%, 98 patients), diabetes mellitus (57.05%, 93 patients), cardiovascular disease (55.21%, 90 patients), chronic obstructive pulmonary disease (46.62%, 76 patients), and chronic kidney disease (47.85%, 78 patients). Other notable comorbidities included Parkinson’s disease (30.67%, 50 patients), dementia (45.39%, 74 patients), osteoporosis (50.30%, 82 patients), hyperlipidemia (11.04%, 18 patients), and stroke history (52.76%, 86 patients). Additionally, anemia was present in 71.77% (117 patients), while depression was reported in 58.28% (95 patients). A variety of other comorbidities, including rheumatoid arthritis, gastrointestinal disorders, chronic pain conditions, sleep apnea, and incontinence, were also prevalent among the study population (Table 2)

**Table 2:** Surgical and Comorbid Conditions Data is presented as frequencies and percentages or means and standard deviation.

Characteristics	Values
Surgical History	
Joint Replacements	15 (9.20%)
No History	132 (80.98%)
Previous Fractures	16 (9.81%)
Surgical Approach	
Non-Surgical (Conservative) Approach	
Casting or Traction	34 (20.85%)
Pain Management and Rehabilitation	36 (21.47%)
Surgical (Operative) Approach	
Hemiarthroplasty	27 (16.56%)
Open Reduction and Internal Fixation (ORIF)	37 (22.69%)
Total Hip Replacement (THR)	29 (17.79%)
Comorbidities	
Hypertension	98 (60.12%)
Diabetes Mellitus	93 (57.05%)
Cardiovascular Disease	90 (55.21%)
Chronic Obstructive Pulmonary Disease	76 (46.62%)
Chronic Kidney Disease	78 (47.85%)
Parkinson’s Disease	50 (30.67%)
Dementia	74 (45.39%)
Osteoporosis	82 (50.30%)
Hyperlipidemia	18 (11.04%)
Stroke History	86 (52.76%)
Previous Cancer	04 (2.45%)
Depression	95 (58.28%)
Anemia	117 (71.77%)
Thyroid Disorders	15 (9.20%)
Rheumatoid Arthritis	23 (14.11%)
Gastrointestinal Disorders	88 (53.98%)
Chronic Pain Conditions	82 (50.30%)
Sleep Apnea	81 (49.69%)
Incontinence	90 (55.21%)
Glaucoma	89 (54.60%)
Hearing Impairment	76 (46.62%)
Hepatitis B	12 (7.36%)
Hepatitis C	08 (4.90%)
Malnutrition	77 (47.23%)

Postoperative outcomes for the 163 patients revealed that 49.07% (80 patients) experienced severe pain, while 46.62% (76 patients) had moderate pain, and 4.29% (seven patients) reported mild pain according to the Visual Analog Scale. Preoperatively, 35.58% (58 patients) had severe disability requiring frequent assistance, 33.74% (55 patients) had mild to moderate disability, and 23.31% (38 patients) were completely dependent on others for assistance. Postoperative complications were reported in a significant portion of patients, with 31.90% (52 patients) experiencing hyperglycemia, 18.40% (30 patients) developing deep vein thrombosis (DVT), and 12.88% (21 patients) suffering from heart failure. Other complications included anxiety and depression (13.49%, 22 patients), malnutrition (4.29%, seven patients), and wound dehiscence (3.06%, five patients). The average length of hospital stay was 16.44 ± 7.42 days, and the average time to surgery was 3.58 ± 1.76 hours. Patients underwent an average of 58.64 ± 21.12 hours of physical therapy, with 57.05% (93 patients) receiving hospital-based rehabilitation and 42.94% (70 patients) having outpatient rehabilitation. Follow-up visits averaged 4.96 ± 2.88, and 41.71% (68 patients) were partially compliant with follow-up care, while 28.22% (46 patients) were fully compliant and 30.06% (49 patients) were non-compliant (Table 3).

**Table 3:** Postoperative Factors and Recovery Outcomes Data is presented as frequencies and percentages or means and standard deviation.

Characteristics	Values
Pain Levels (Visual Analog Scale)	
Mild Pain	07 (4.29%)
Moderate Pain	76 (46.62%)
Severe Pain	80 (49.07%)
Preoperative Functional Status Barthel Index Category	
Complete Dependence (Total Assistance)	38 (23.31%)
Independent (Minimal Assistance)	12 (7.63%)
Mild to Moderate Disability (Some Assistance)	55 (33.74%)
Severe Disability (Frequent Assistance)	58 (35.58%)
Postoperative Complications	
None	47 (28.83%)
Heart Failure	21 (12.88%)
Hyperglycemia	52 (31.90%)
Deep Vein Thrombosis	30 (18.40%)
Stroke	06 (3.68%)
Anxiety and Depression	22 (13.49%)
Sepsis	14 (8.58%)
Delirium	08 (4.90%)
Urinary Retention	06 (3.68%)
Postoperative Pain Syndrome	09 (5.52%)
Pneumonia	02 (1.22%)
Wound Dehiscence	05 (3.06%)
Malnutrition	07 (4.29%)
Fat Embolism	02 (1.22%)
Malunion	12 (7.36%)
Hemarthrosis	02 (1.22%)
Arrhythmias	01 (0.61%)
Hypoglycemia	01 (0.61%)
Ileus	02 (1.22%)
Aspiration Pneumonia	01 (0.61%)
Length of Hospital Stay (days)	16.44±7.42
Time to Surgery (hours)	3.58±1.76
Duration of Physical Therapy (hours)	58.64±21.12
Type of Rehabilitation	
Hospital-based	93 (57.05%)
Outpatient	70 (42.94%)
Follow-up Visits	4.96±2.88
Follow-up Care Compliance	
Fully Compliant	46 (28.22%)
Non-Compliant	49 (30.06%)
Partially Compliant	68 (41.71%)

**Figure 1:**
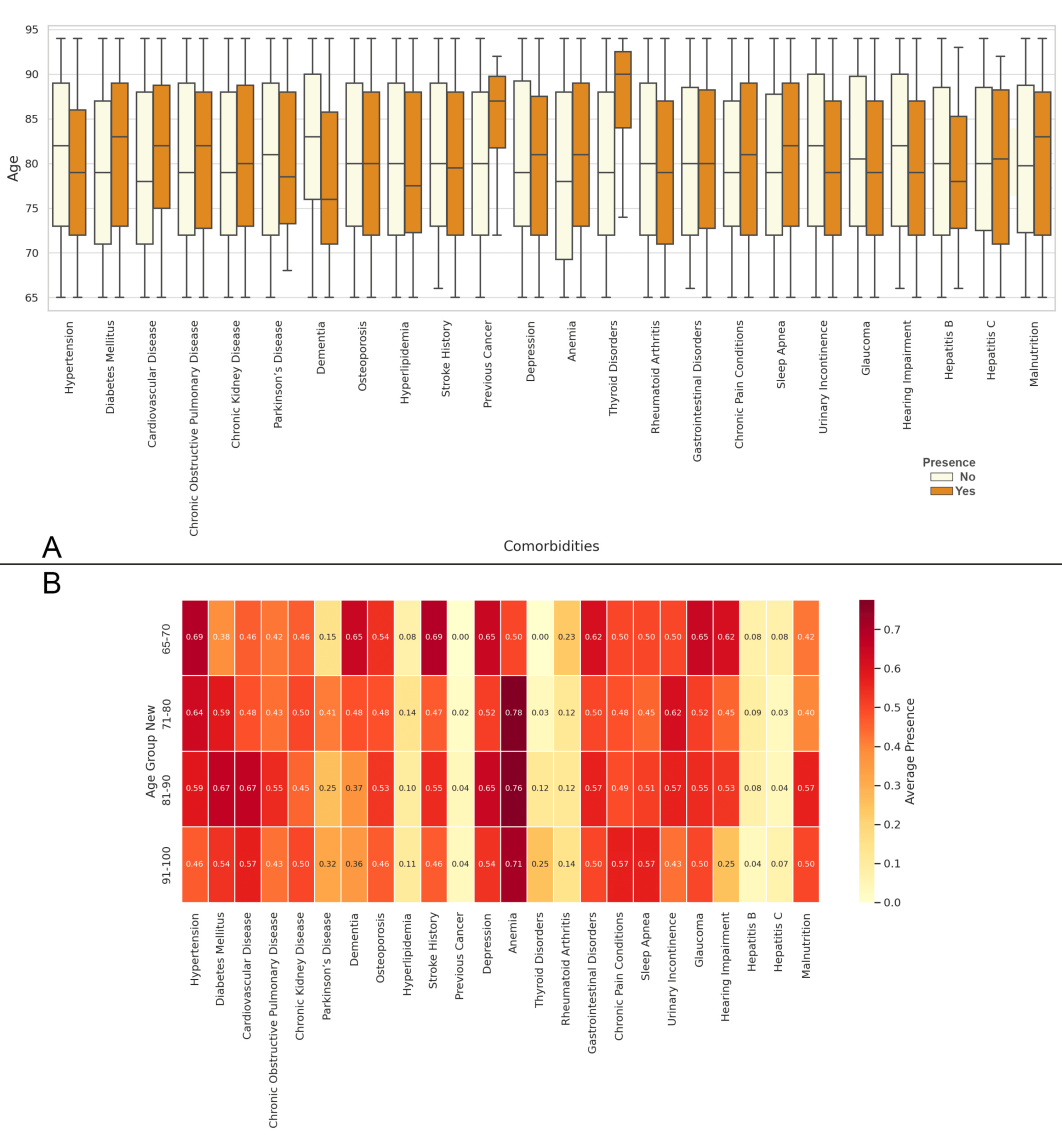
Relationship between Comorbidities and Age Figure A: Age Distribution and Prevalence of Comorbidities in Elderly Patients with Neck of Femur Fractures Figure B: Correlation Matrix of Age Groups and Comorbidities in Elderly Patients with Neck of Femur Fractures

In Figure 1A, the relationship between age and comorbidities indicates that older patients, particularly those in the 85-90 years range, tend to have a higher prevalence of conditions such as thyroid disorder, previous cancer, diabetes, and malnutrition, among others. In Figure 1B, the correlation matrix shows the relationships between age groups and comorbidities. In the 65-70 years age group, the highest correlation is with stroke history (0.69), indicating a strong association between stroke and this age group. For the 71-80 years group, anemia shows the highest correlation (0.78). Moving to the 81-90 years group, anemia remains the highest correlated comorbidity (0.76), though other conditions like cardiovascular diseases (0.67) and diabetes (0.67) also show strong correlations. In the 91-100 years group, the highest correlation is again with anemia (0.71), followed by cardiovascular diseases and chronic pain conditions (0.57) (Figure 1).

**Figure 2:**
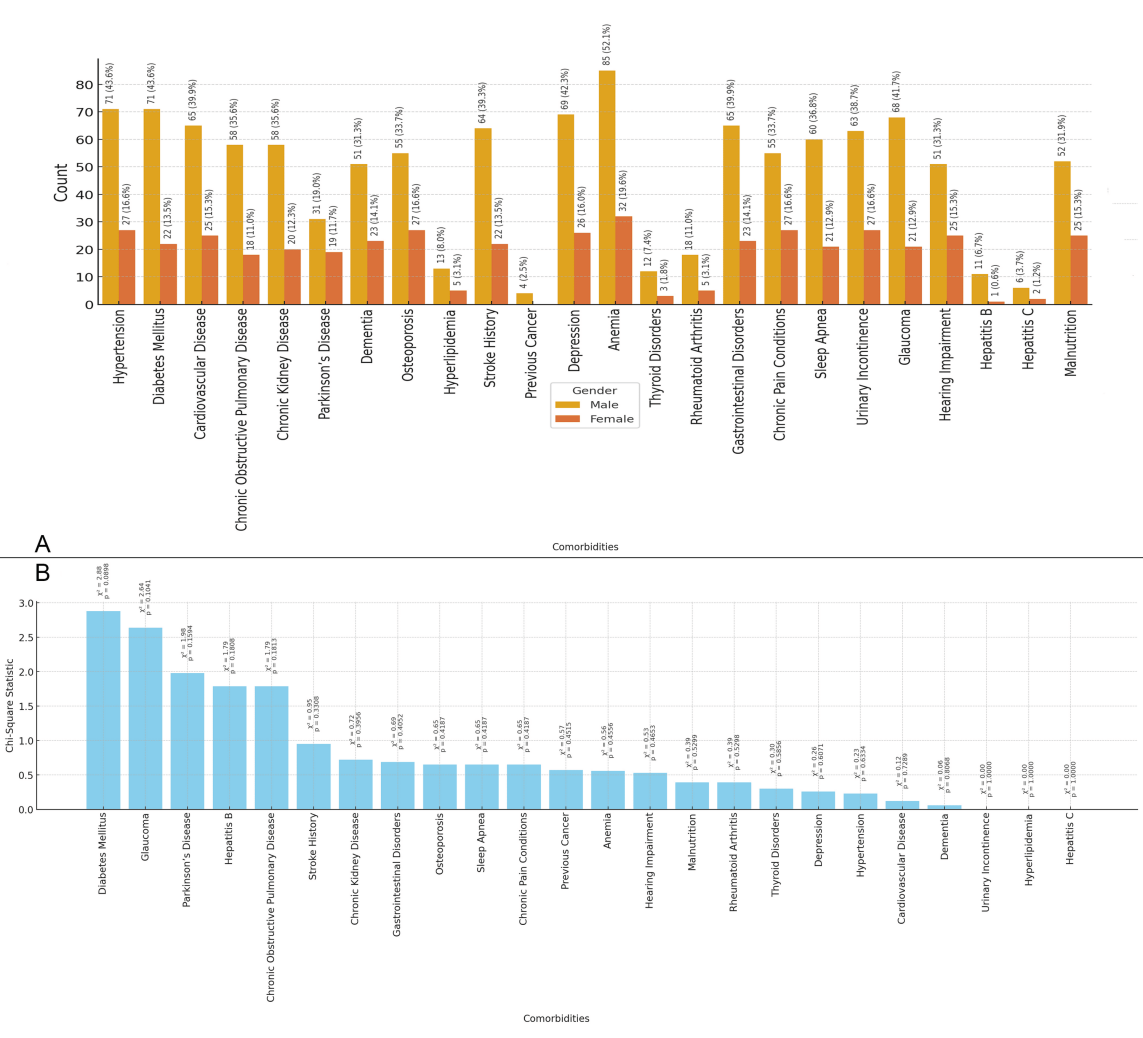
Relationship between Comorbidities and Gender Figure A: Gender Distribution of Comorbidities in Elderly Patients with Neck of Femur Fractures Figure B: Chi-Square Analysis of Gender and Comorbidities in Elderly Patients with Neck of Femur Fractures

In Figure 2A, the bar chart illustrates the gender distribution of various comorbidities in the study population. The most prevalent comorbidities were anemia (85 males, 52.1% and 32 females, 19.6%), hypertension (71 males, 43.6% and 27 females, 16.6%), diabetes mellitus (71 males, 43.6% and 22 females, 13.5%), and cardiovascular disease (65 males, 39.9% and 25 females, 15.3%), with higher counts observed in males for most conditions. Figure 2B presents the chi-square statistic for each comorbidity. No chi-square values were significant, suggesting that gender does not play a significant role in the presence of these comorbidities in relation to neck of femur fractures (Figure 2).

**Figure 3:**
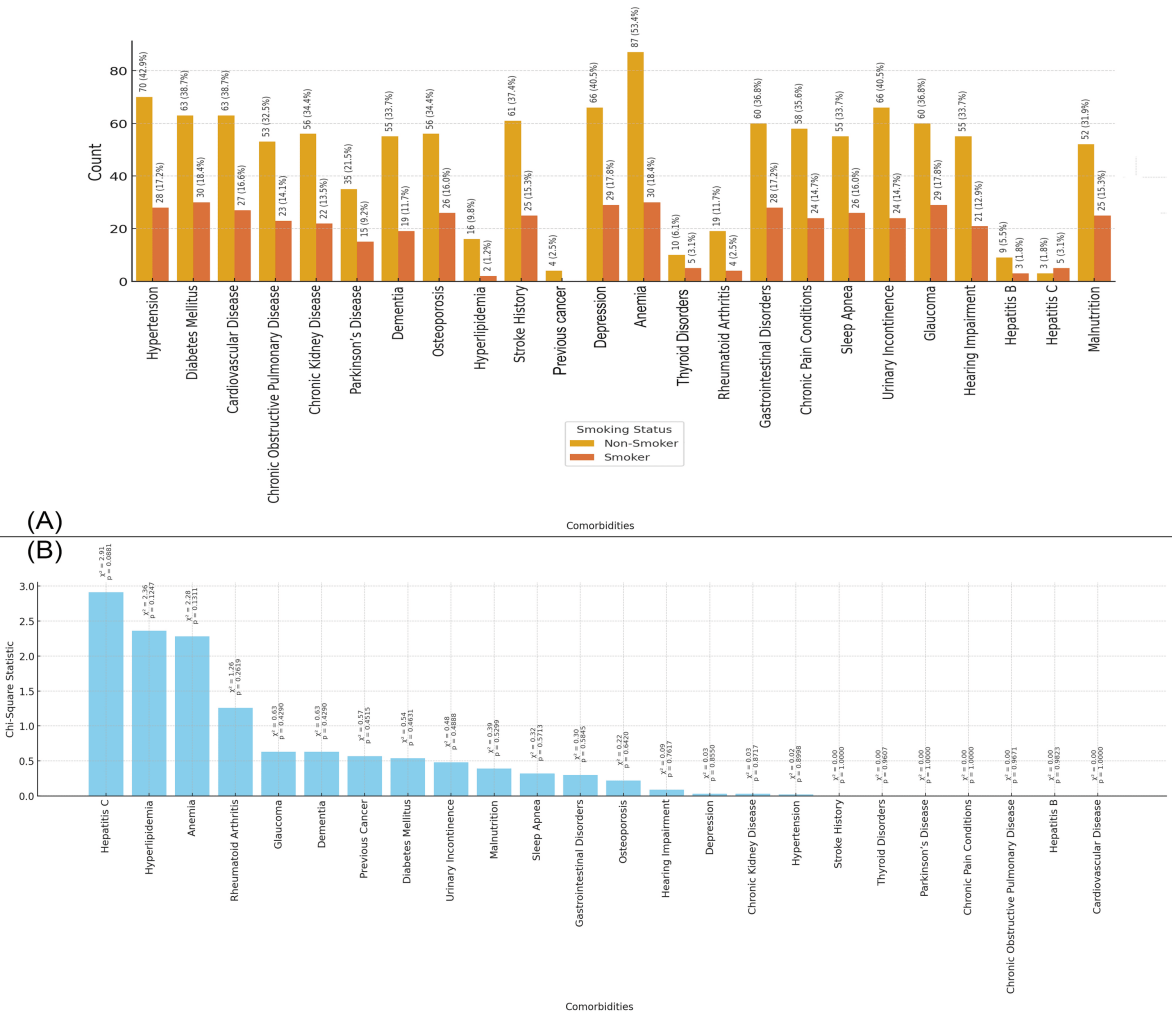
Relationship between Comorbidities and Smoking status Figure A: Smoking Status and  Distribution of Comorbidities in Elderly Patients with Neck of Femur Fractures Figure B: Chi-Square Analysis of Smoking Status and Comorbidities in Elderly Patients with Neck of Femur Fractures

In Figure 3A, the bar chart shows the distribution of comorbidities in the study population based on smoking status. Smokers have a higher prevalence of conditions such as diabetes mellitus (30 patients, 18.4%) and hypertension (28 patients, 17.2%). The chart also highlights the prevalence of other comorbidities like cardiovascular disease, chronic kidney disease, and COPD in the smokers group, among others. Figure 3B presents the chi-square statistics for the relationship between smoking status and each comorbidity. While some comorbidities show a higher prevalence in smokers, no chi-square values were statistically significant, suggesting that smoking status does not significantly affect the presence of these comorbidities in the study population (Figure 3).

**Figure 4:**
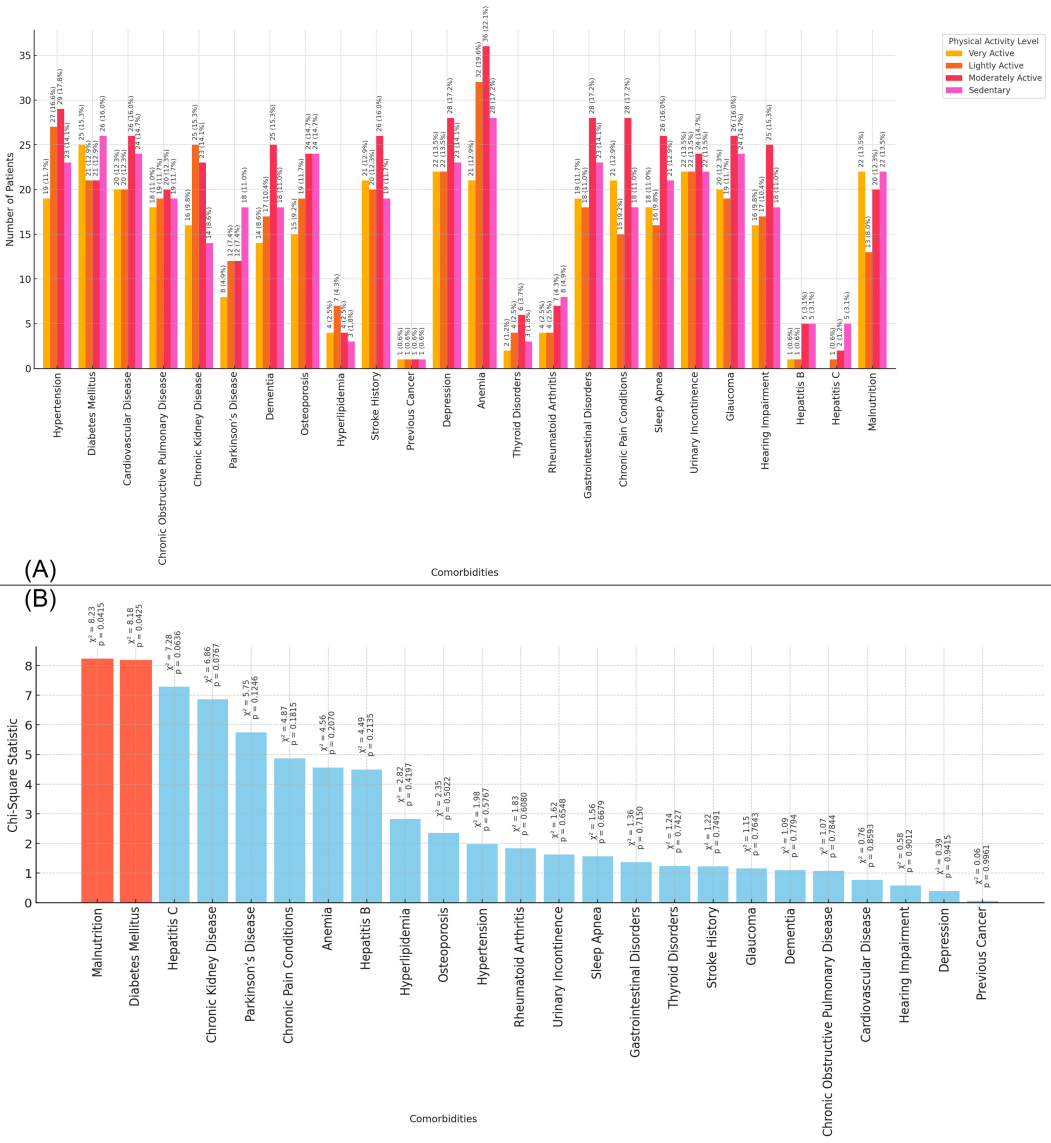
Relationship between Comorbidities and Physical Activity Levels Figure A: Physical Activity Levels and Distribution of Comorbidities in Elderly Patients with Neck of Femur Fractures Figure B: Chi-Square Analysis of Physical Activity Levels and Comorbidities in Elderly Patients with Neck of Femur Fractures

In Figure 4A, the bar chart shows the distribution of comorbidities in the study population based on physical activity levels. Sedentary patients exhibit a higher prevalence of conditions like anemia (28 patients, 17.2%), diabetes mellitus (26 patients, 16.0%), and cardiovascular diseases (24 patients, 17.7%), while very active patients show a lower prevalence across most comorbidities. Moderately active patients have a relatively balanced distribution of comorbidities, with notable prevalence of anemia (36 patients, 22.1%) and hypertension (29 patients, 17.8%). Lightly active patients also show a higher prevalence of conditions such as anemia (32 patients, 19.6%) and hypertension (29 patients, 17.8%). Figure 4B presents the chi-square statistics for the relationship between physical activity level and each comorbidity. The chi-square values for malnutrition (chi-square = 8.23, p = 0.041) and diabetes mellitus (chi-square = 8.18, p = 0.042) are the highest, indicating significant associations between physical activity levels and these conditions (Figure 4).

**Figure 5:**
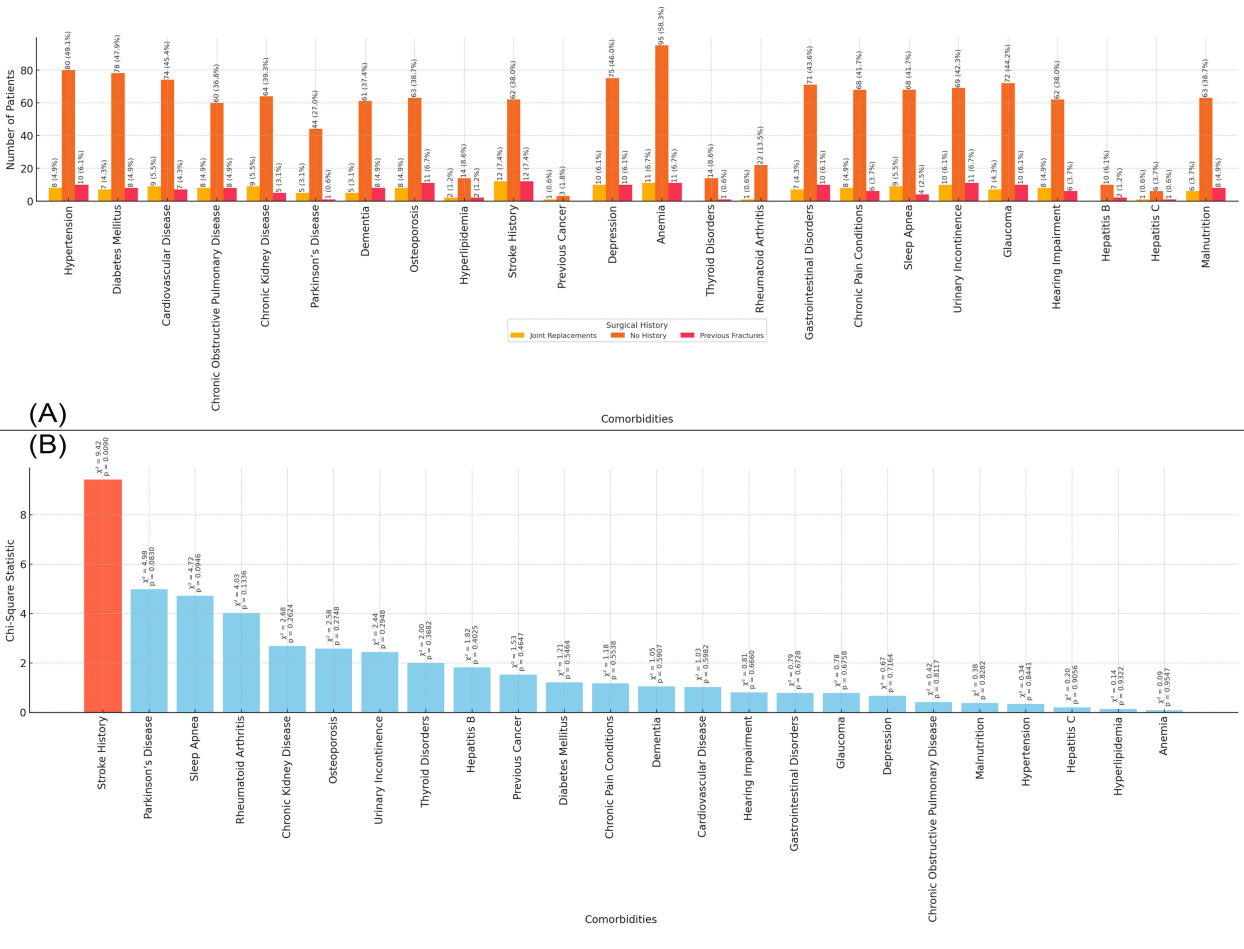
Relationship between Comorbidities and Surgical History Figure A: Surgical History and Distribution of Comorbidities in Elderly Patients with Neck of Femur Fractures Figure B: Chi-Square Analysis of Surgical History and Comorbidities in Elderly Patients with Neck of Femur Fractures

In Figure 5A, the bar chart shows the distribution of comorbidities in the study population based on surgical history. Patients with a previous fracture history exhibit a higher prevalence of conditions like hypertension (10 patients, 6.1%), diabetes mellitus (eight patients, 4.9%), and stroke history (12 patients, 7.4%), while those with joint replacements show a higher prevalence of anemia (11 patients, 6.7%) and stroke (12 patients, 7.4%). The chart highlights the differences in comorbidity prevalence between those with no surgical history and those with prior fractures or joint replacements. Figure 5B presents the chi-square statistics for the relationship between surgical history and each comorbidity. The highest chi-square value is for stroke history (chi-square = 9.42, p-value = 0.009), indicating a significant association between surgical history and the prevalence of stroke in this population (Figure 5).

**Figure 6:**
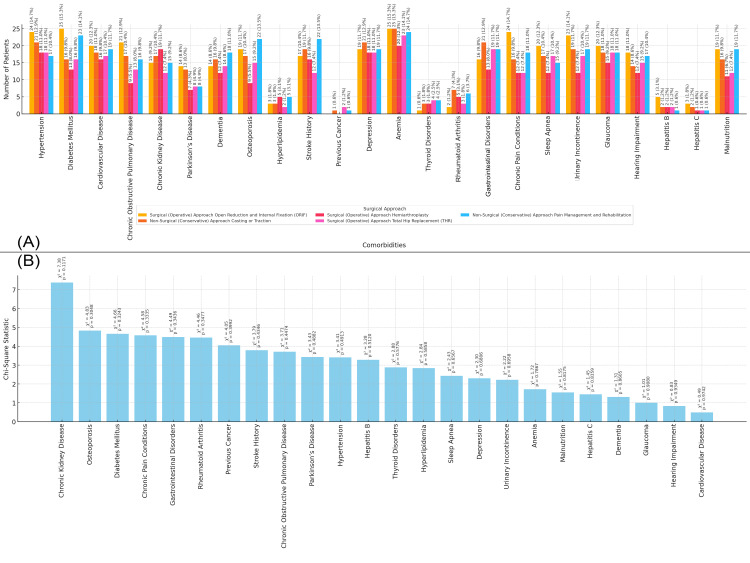
Relationship between Comorbidities and Surgical Approach Figure A: Distribution of Comorbidities by Surgical Approach in Elderly Patients with Neck of Femur Fractures Figure B: Chi-Square Analysis of Surgical Approach and Comorbidities in Elderly Patients with Neck of Femur Fractures

In Figure 6A, the bar chart shows the distribution of comorbidities in the study population based on the type of surgical approach. Patients who underwent open reduction and internal fixation (ORIF) exhibit a higher prevalence of conditions such as anemia (25 patients, 15.3%), diabetes mellitus (25 patients, 15.3%), and hypertension (24 patients, 14.7%). Those who underwent hemiarthroplasty show a higher prevalence of anemia (20 patients, 12.3%) and hypertension (18 patients, 11.0%). Patients who received non-surgical approaches, such as casting and traction or pain management and rehabilitation, have a higher prevalence of anemia (24 patients, 17.7%) and diabetes mellitus (23 patients, 14.1%). Figure 6B presents the chi-square statistics for the relationship between surgical approach and each comorbidity. No significant p-values were found, suggesting that the surgical approach does not have a significant impact on the presence of these comorbidities in the study population (Figure 6).

**Figure 7:**
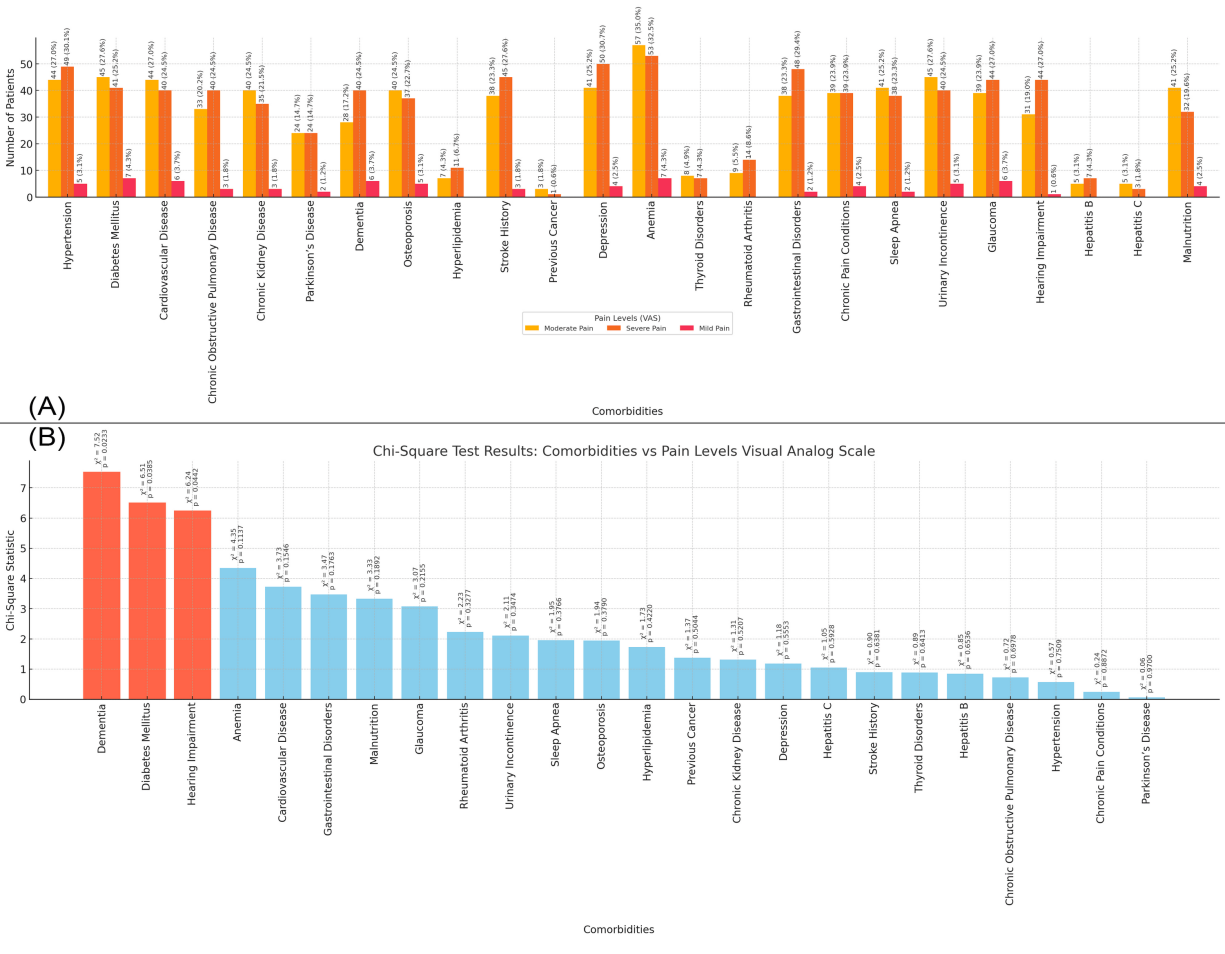
Relationship between Comorbidities and Pain Levels Measured by the Visual Analog Scale Figure A: Distribution of Comorbidities by Pain Levels in Elderly Patients with Neck of Femur Fractures Figure B: Chi-Square Test Results: Comorbidities vs Pain Levels Measured by the Visual Analog Scale

In Figure 7A, the bar chart shows the distribution of comorbidities in the study population based on pain levels as measured by the Visual Analog Scale (VAS). Patients experiencing severe pain exhibit a higher prevalence of conditions such as anemia (53 patients, 32.5%), hypertension (49 patients, 30.1%), and depression (50 patients, 30.7%). Moderate pain is associated with conditions like anemia (57 patients, 35.0%), cardiovascular disease (44 patients, 27.0%), hypertension (44 patients, 27.0%), and diabetes (45 patients, 27.6%), while mild pain shows a lower prevalence across most comorbidities. Figure 7B presents the chi-square statistics for the relationship between comorbidities and pain levels. The highest chi-square values are for dementia (chi-square = 7.52, p-value = 0.023), diabetes mellitus (chi-square = 6.51, p-value = 0.038), and hearing impairment (chi-square = 6.24, p-value = 0.044), indicating significant associations between these conditions and higher pain levels in the study population (Figure 7).

**Figure 8:**
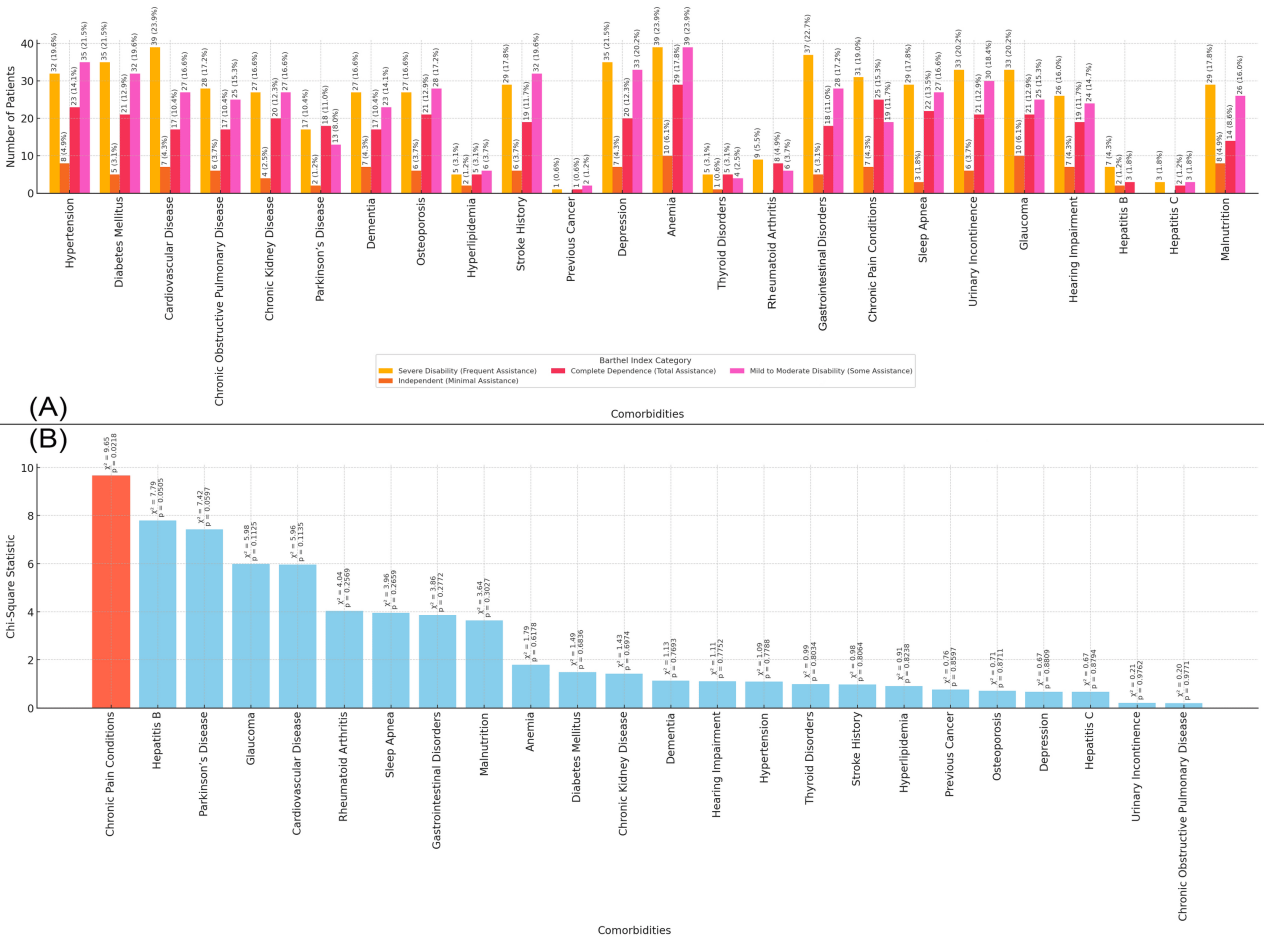
Relationship between Comorbidities and the Barthel Index Figure A: Distribution of Comorbidities Based on the Barthel Index Categories in Elderly Patients with Neck of Femur Fractures Figure B: Chi-Square Analysis of Comorbidities and Barthel Index Categories in Elderly Patients with Neck of Femur Fractures

In Figure 8A, the bar chart shows the distribution of comorbidities in the study population based on Barthel Index categories, which assess the level of assistance required. Severe disability (frequent assistance) is most prevalent among patients with conditions like anemia and cardiovascular diseases (39 patients, 23.9%), gastrointestinal disorders (37 patients, 22.7%), and diabetes mellitus and depression (35 patients, 21.5%). Independent (minimal assistance) patients show a higher prevalence of conditions such as anemia and glaucoma (10 patients, 6.1%), and hypertension and malnutrition (eight patients, 4.9%). Patients classified with mild to moderate disability (some assistance) have a balanced distribution of conditions like anemia (39 patients, 23.9%), hypertension (35 patients, 21.5%), and diabetes mellitus (32 patients, 19.6%). Figure 8B presents the chi-square statistics for the relationship between comorbidities and Barthel Index categories. The highest chi-square value is for chronic pain conditions (chi-square = 9.65, p-value = 0.021), suggesting a significant association between these comorbidities and the need for frequent assistance in daily activities (Figure 8).

**Figure 9:**
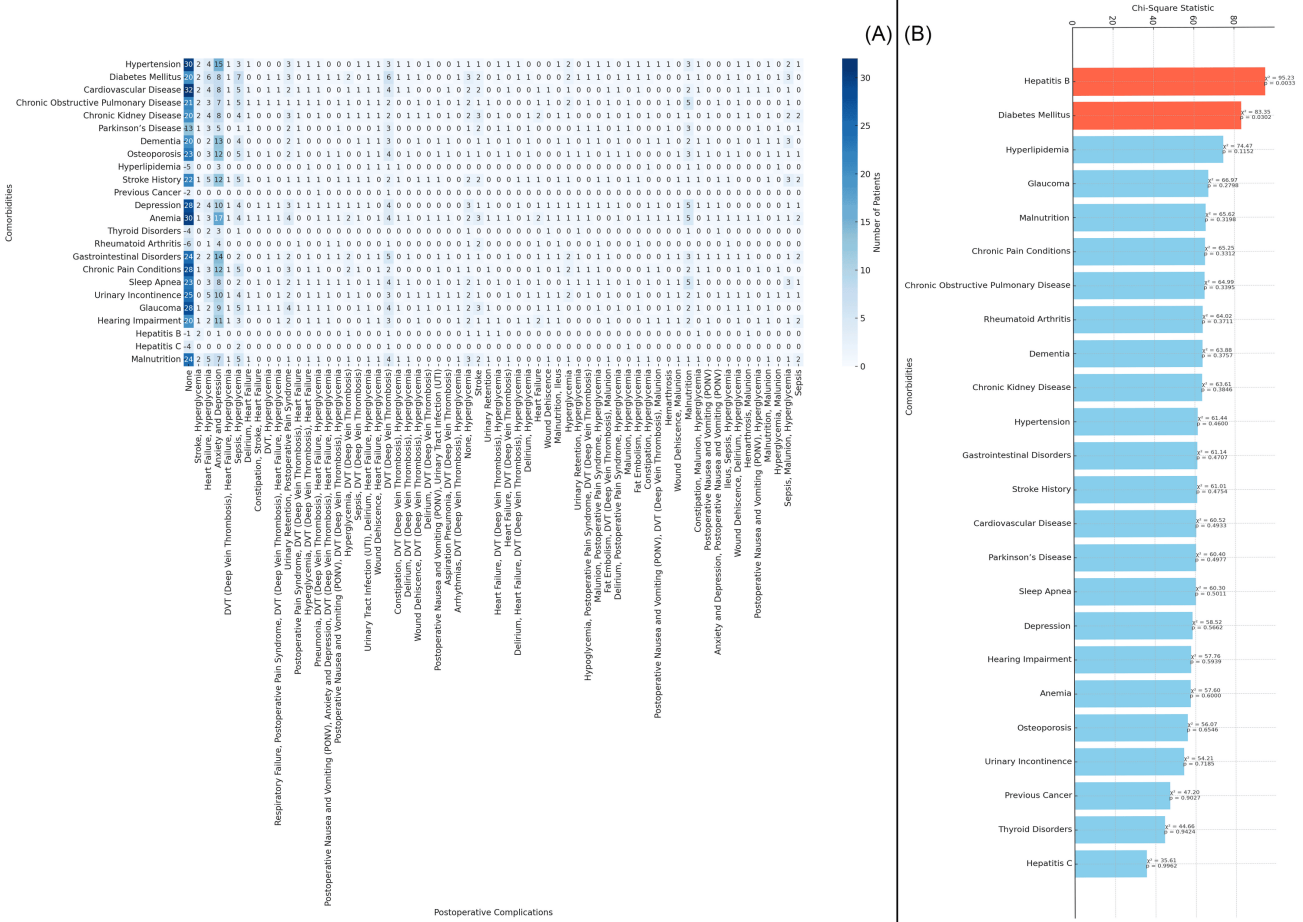
Relationship between Comorbidities and Postoperative Complications Figure A: Distribution of Postoperative Complications Based on Comorbidities in Elderly Patients with Neck of Femur Fractures Figure B: Chi-Square Analysis of Comorbidities and Postoperative Complications in Elderly Patients with Neck of Femur Fractures

In Figure 9A, the heatmap illustrates the distribution of postoperative complications across various comorbidities in the study population. The number of patients experiencing each complication is represented by varying shades of blue, with darker blue indicating higher numbers of patients. Anxiety and depression, heart failure, hyperglycemia, deep vein thrombosis, and malnutrition are among the conditions most frequently associated with postoperative complications. Figure 9B presents the chi-square statistics for the relationship between each comorbidity and postoperative complications. The highest chi-square values are observed for hepatitis B (chi-square = 95.23, p-value = 0.003) and diabetes mellitus (chi-square = 83.35, p-value = 0.030), indicating significant associations between these comorbidities and the occurrence of postoperative complications in elderly patients with neck of femur fractures (Figure 9).

**Figure 10:**
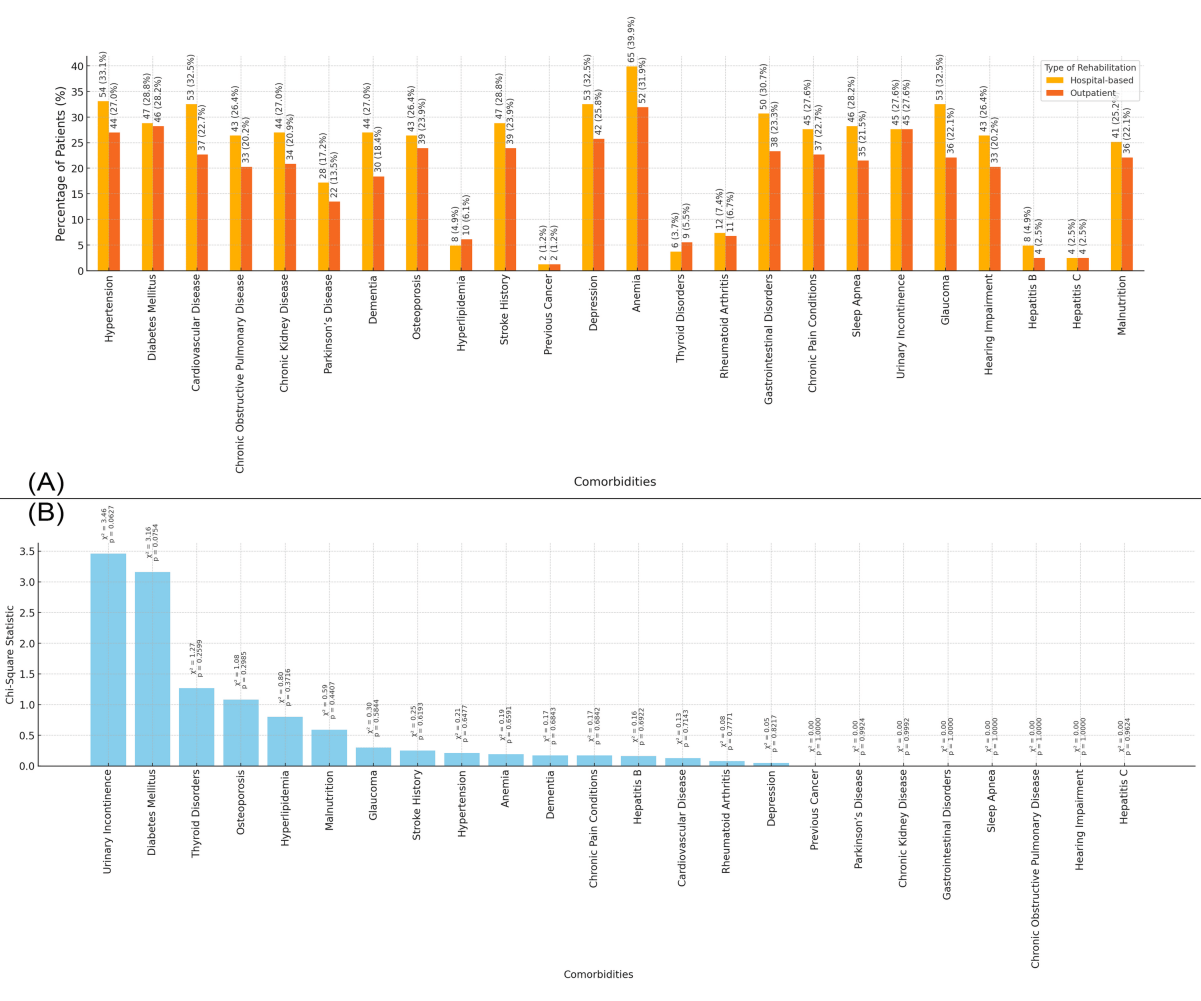
Relationship between Comorbidities and Type of Rehabilitation Figure A: Distribution of Comorbidities Based on Type of Rehabilitation in Elderly Patients with Neck of Femur Fractures Figure B: Chi-Square Analysis of Comorbidities and Type of Rehabilitation in Elderly Patients with Neck of Femur Fractures

In Figure 10A, the bar chart shows the distribution of comorbidities in the study population based on the type of rehabilitation received. Patients who received hospital-based rehabilitation exhibit a higher prevalence of conditions such as anemia (65 patients, 39.95%), hypertension (54 patients, 33.1%), and cardiovascular disease (53 patients, 32.2%). In contrast, patients receiving outpatient rehabilitation show a higher prevalence of conditions like anemia (52 patients, 31.9%), diabetes (46 patients, 28.2%), and hypertension (44 patients, 27.0%). Figure 10B presents the chi-square statistics for the relationship between comorbidities and the type of rehabilitation. No statistically significant p-values were observed, indicating no strong association between comorbidities and the type of rehabilitation received (Figure 10).

**Figure 11:**
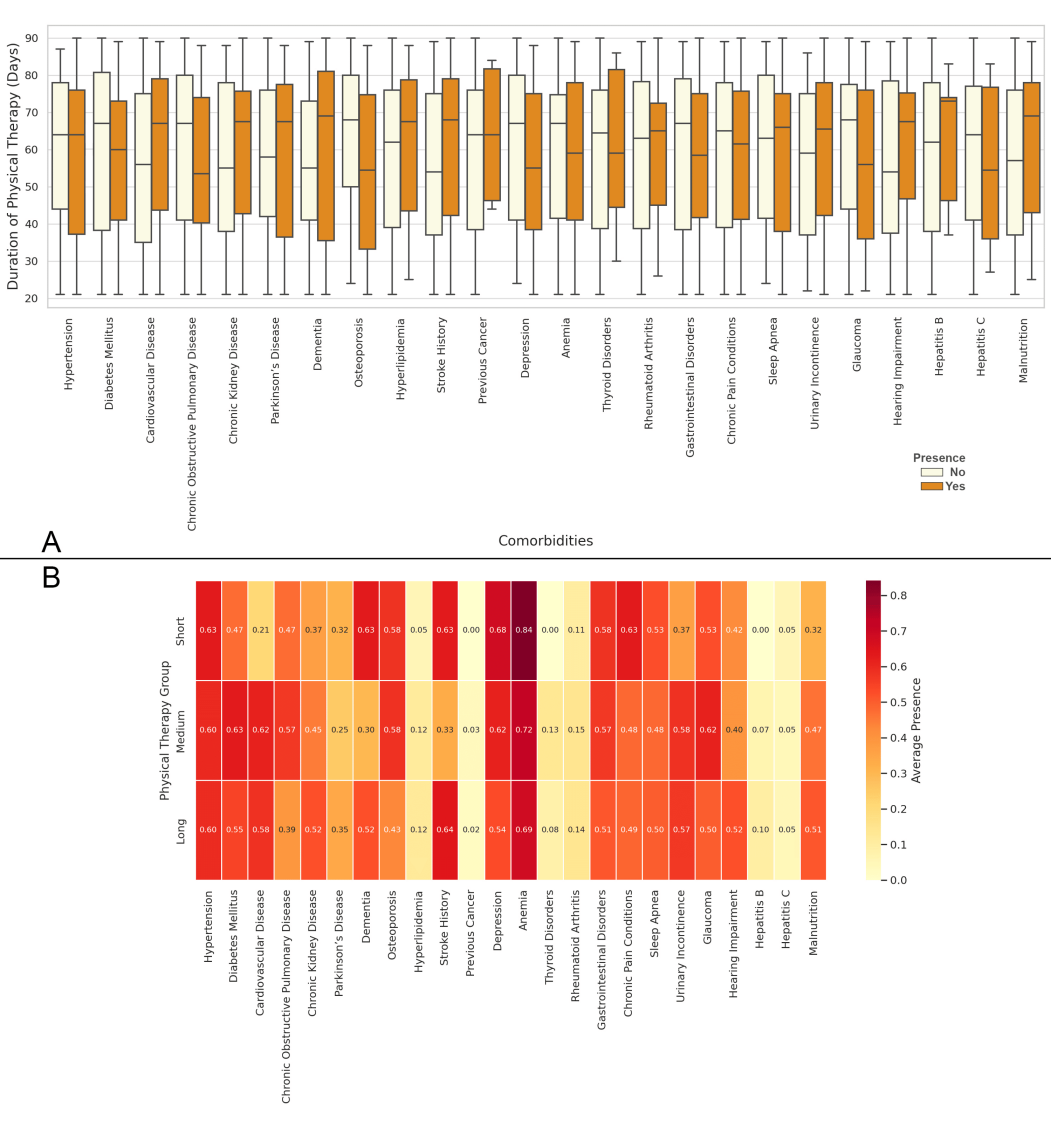
Relationship between Comorbidities and the Duration of Physical Therapy Figure A: Distribution of Duration of Physical Therapy Based on the Presence of Comorbidities in Elderly Patients with Neck of Femur Fractures Figure B: Correlation Matrix of Comorbidities and Duration of Physical Therapy in Elderly Patients with Neck of Femur Fractures Physical therapy duration Groups: Short: Duration ≤ 30 days; Medium: 31 ≤ Duration ≤ 60 days; Long: Duration > 60 days

In Figure 11A, the boxplot shows the duration of physical therapy based on the presence or absence of various comorbidities. Patients with conditions such as hepatitis B, cardiovascular disease, stroke history, and malnutrition tend to have a longer duration of physical therapy. Figure 11B presents the correlation matrix between comorbidities and the duration of physical therapy. The highest positive correlations are observed with anemia, stroke, and hypertension, suggesting that these comorbidities are strongly associated with longer durations of physical therapy. Conversely, conditions like hepatitis B and hepatitis C show much lower correlations, indicating that these comorbidities have a less significant impact on the duration of physical therapy (Figure 11).

**Figure 12:**
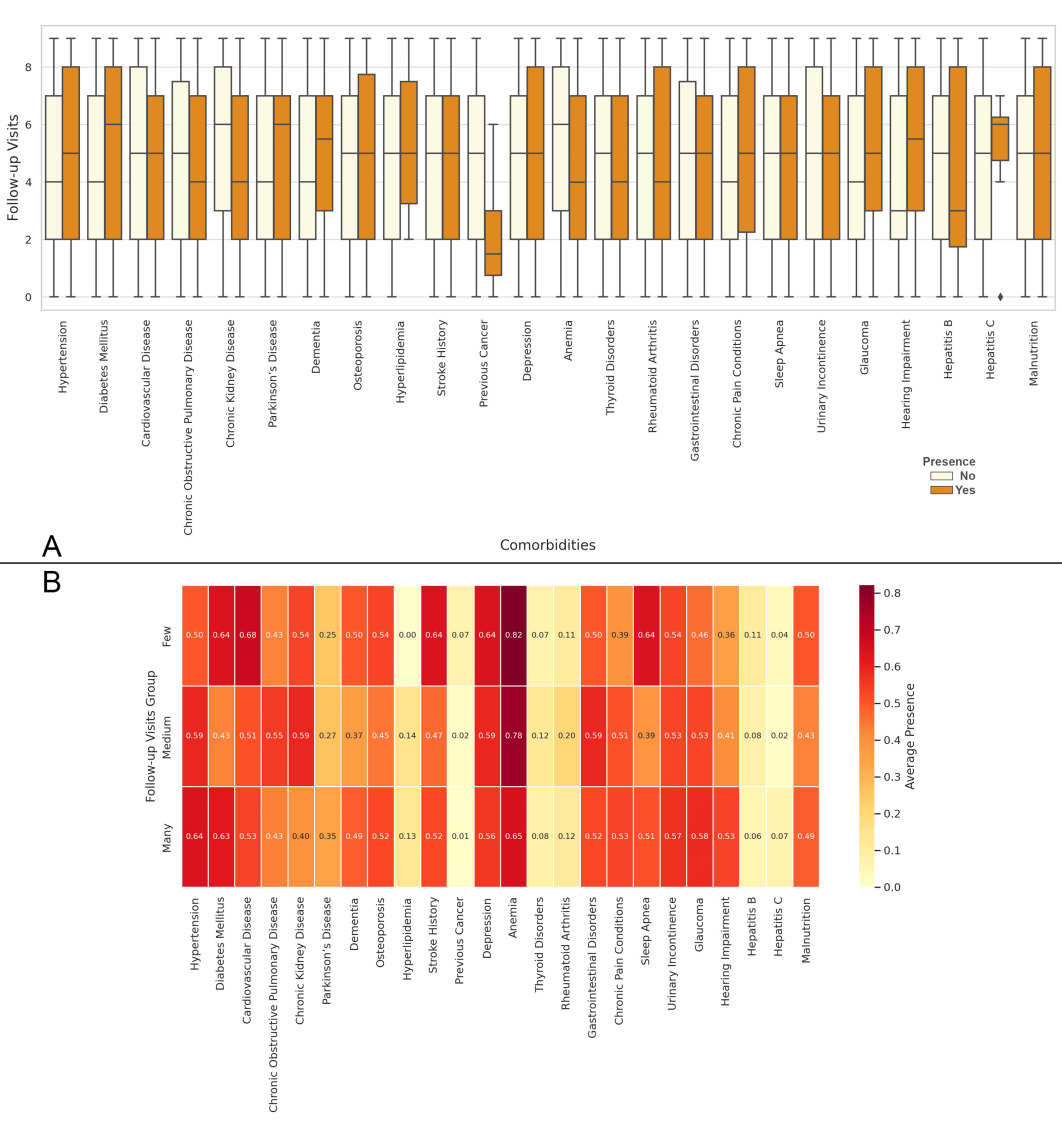
Relationship between Comorbidities and the Number of Follow-up Visits Figure A: Distribution of Follow-up Visits Based on the Presence of Comorbidities in Elderly Patients with Neck of Femur Fractures Figure B: Correlation Matrix of Comorbidities and Follow-up Visits in Elderly Patients with Neck of Femur Fractures Follow-up Visits Groups: Few: 0-2 visits; Medium: 3-5 visits; Many: 6 or more visits

In Figure 12A, the boxplot shows the distribution of follow-up visits based on the presence or absence of various comorbidities. Patients with comorbidities like hypertension, Parkinson's disease, and cardiovascular disease tend to have a higher number of follow-up visits. Figure 12B presents the correlation matrix between the presence of comorbidities and the number of follow-up visits. The highest positive correlations are observed with anemia, diabetes, and hypertension, indicating that these comorbidities are strongly associated with more frequent follow-up visits. Conversely, conditions like hepatitis C and hepatitis B show much lower correlations, suggesting less frequent follow-up visits for patients with these comorbidities (Figure 12).

**Figure 13:**
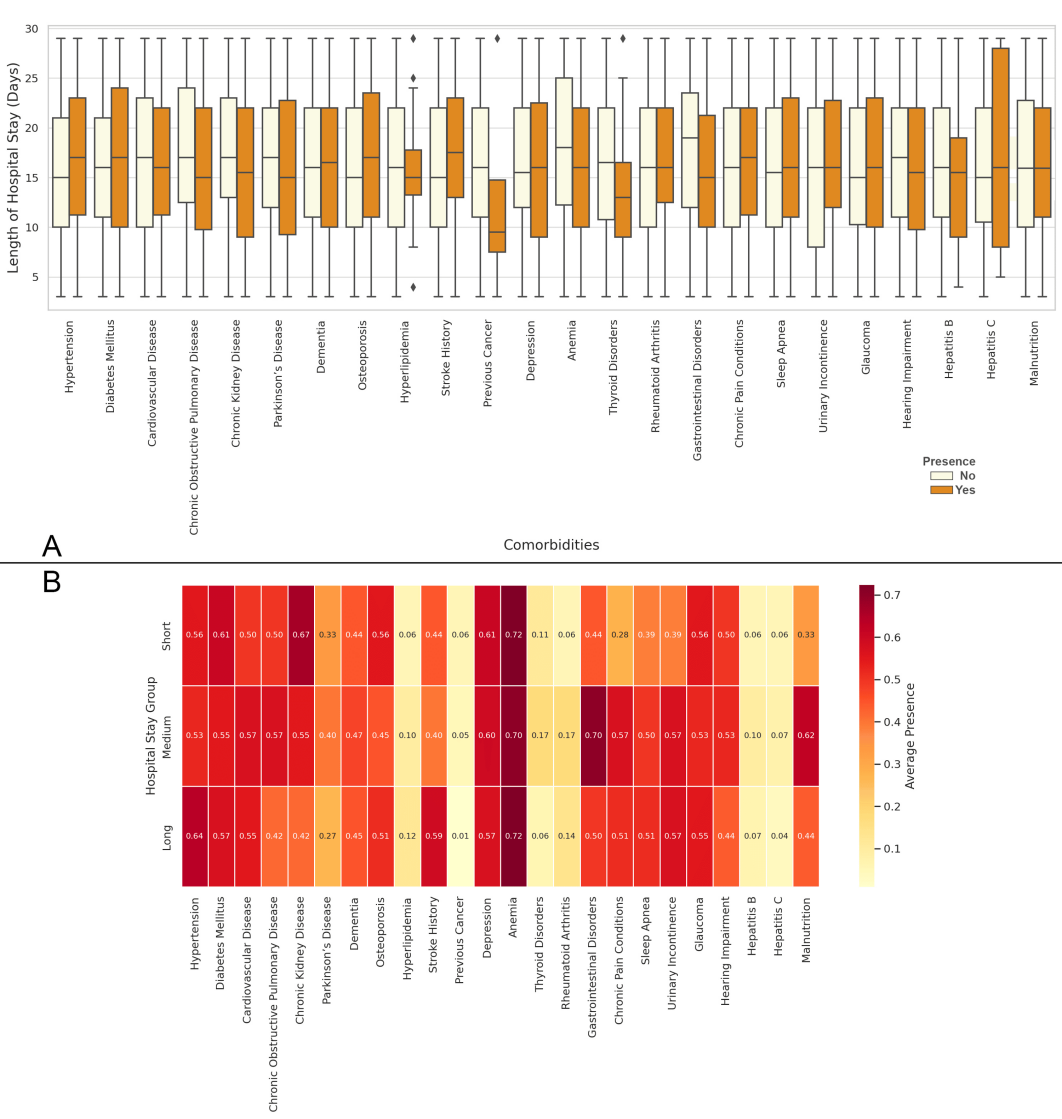
Relationship between Comorbidities and the Length of Hospital Stay Figure A: Length of Hospital Stay Based on the Presence of Comorbidities in Elderly Patients with Neck of Femur Fractures Figure B: Correlation Matrix of Comorbidities and Length of Hospital Stay in Elderly Patients with Neck of Femur Fractures Groups based on hospital stay lengths: Short: Length ≤ 7 days; Medium: 8 ≤ Length ≤ 14 days; Long: Length > 14 days

In Figure 13A, the boxplot shows the length of hospital stay based on the presence or absence of various comorbidities. Patients with conditions such as stroke, diabetes mellitus, cardiovascular disease, and hypertension tend to have longer hospital stays, with stroke showing the highest median length of stay. Figure 13B presents the correlation matrix between comorbidities and the length of hospital stay. The highest positive correlations are observed with anemia, hypertension, and diabetes mellitus, indicating that these comorbidities are strongly associated with longer hospital stays. Conversely, conditions like hepatitis B and hepatitis C show much lower correlations, suggesting these comorbidities have a less significant impact on the length of hospital stay (Figure 13).

**Figure 14:**
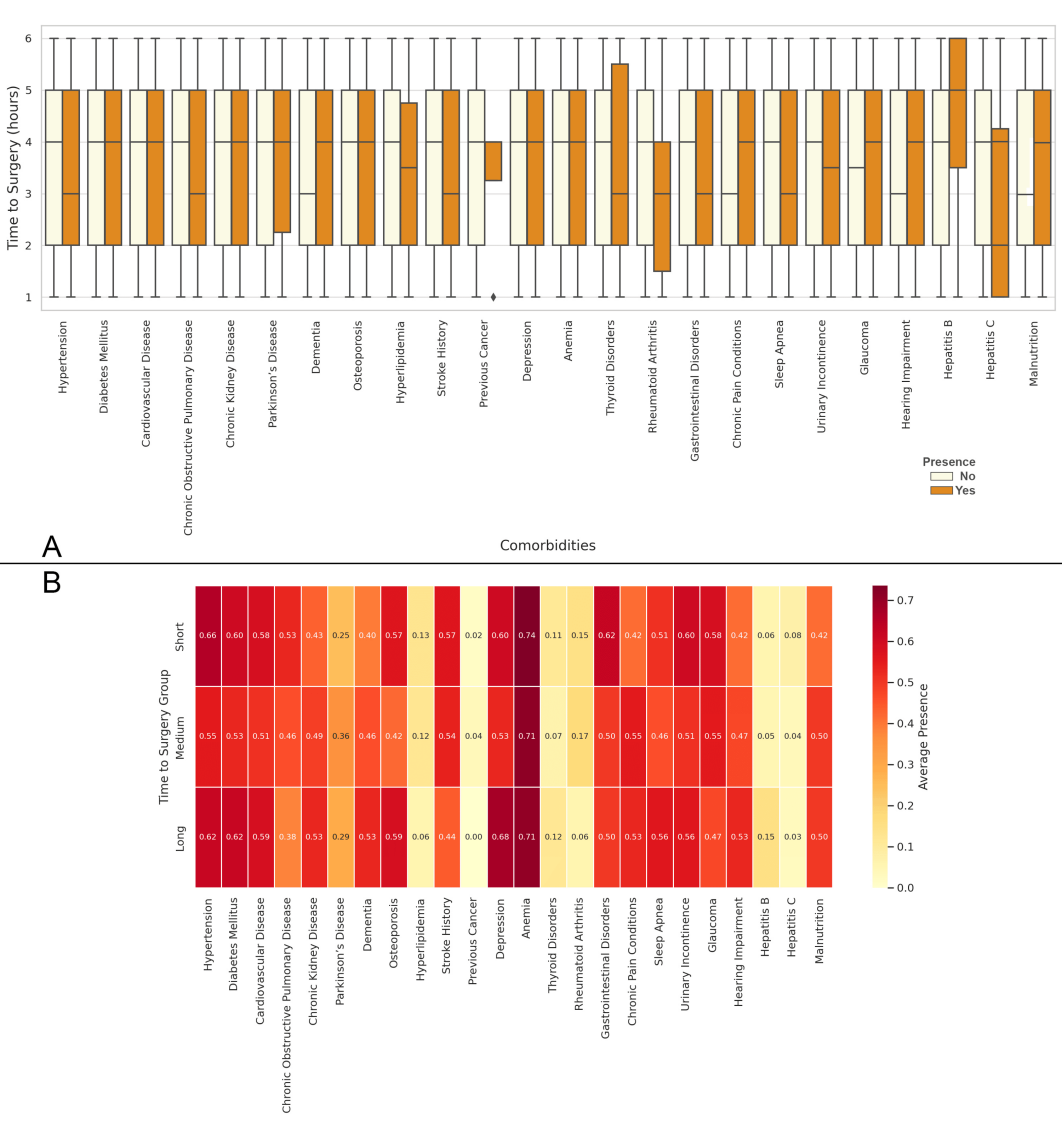
Relationship between Comorbidities and the Time to Surgery Figure A: Distribution of Time to Surgery Based on the Presence of Comorbidities in Elderly Patients with Neck of Femur Fractures Figure B: Correlation Matrix of Comorbidities and Time to Surgery in Elderly Patients with Neck of Femur Fractures Time to surgery groups as follows: Short: Time to surgery ≤ 3 hours; Medium: 4 ≤ Time to surgery ≤ 6 hours; Long: Time to surgery > 6 hours

In Figure 14A, the boxplot shows the time to surgery based on the presence or absence of various comorbidities. Patients with conditions such as hypertension, diabetes mellitus, cardiovascular disease, and depression tend to experience longer delays before surgery. Figure 14B presents the correlation matrix between comorbidities and time to surgery. The highest positive correlations are seen with anemia, hypertension, diabetes mellitus, and depression, indicating that these comorbidities are strongly associated with longer delays in surgery. In contrast, conditions like hepatitis B and hepatitis C show much lower correlations, suggesting these comorbidities have a less significant impact on the time to surgery (Figure 14).

**Figure 15:**
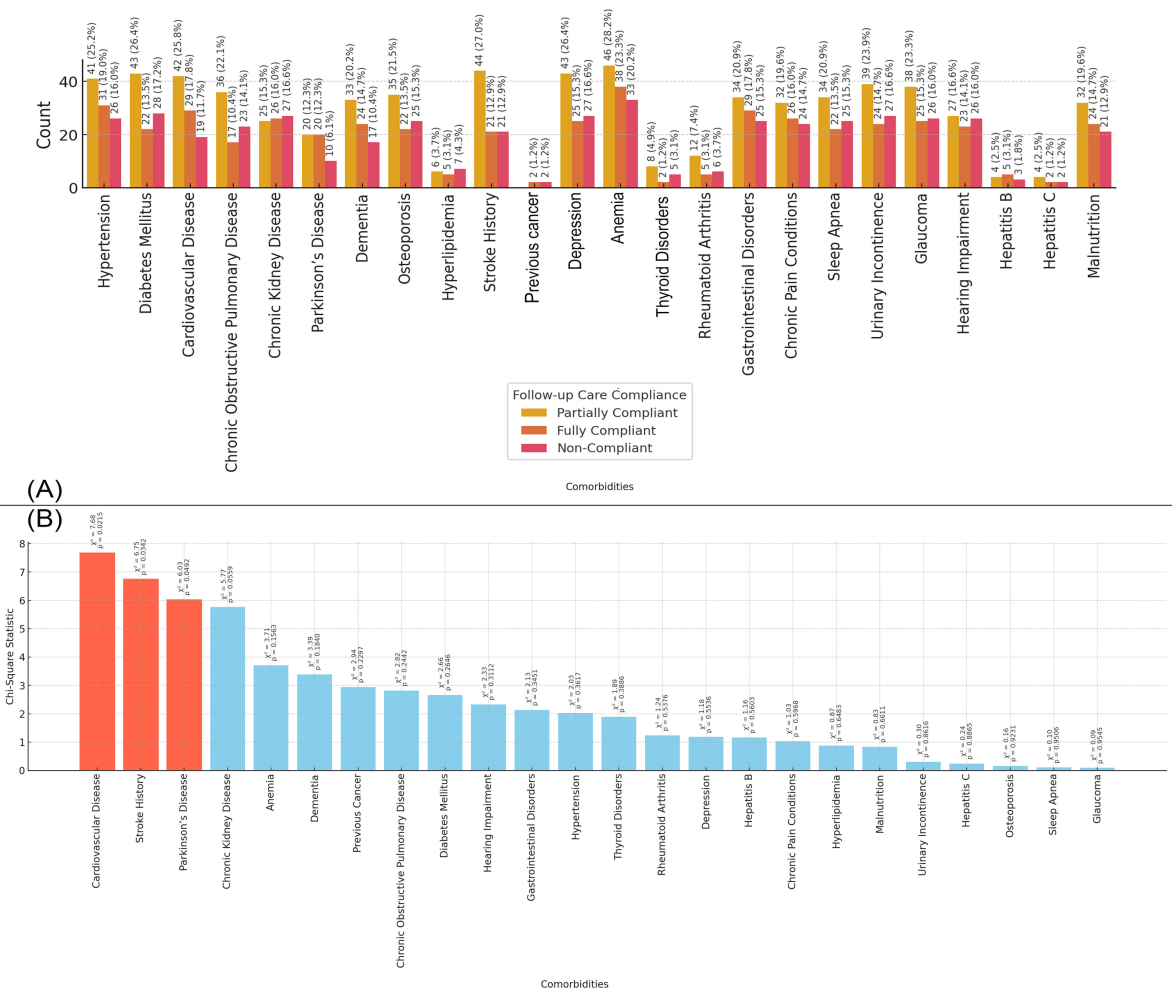
Relationship between Comorbidities and Follow-up Care Compliance Figure A: Distribution of Follow-up Care Compliance Based on Comorbidities in Elderly Patients with Neck of Femur Fractures Figure B: Chi-Square Analysis of Comorbidities and Follow-up Care Compliance in Elderly Patients with Neck of Femur Fractures

In Figure 15A, the bar chart shows the distribution of follow-up care compliance based on the presence or absence of various comorbidities. Patients with conditions such as anemia (33 patients, 20.2%), hypertension (26 patients, 16.0%), and diabetes mellitus (28 patients, 17.2%) tend to have higher rates of non-compliance with follow-up care, while those with anemia (46 patients, 28.2%), stroke (43 patients, 26.4%), and diabetes mellitus (43 patients, 26.4%) show a higher proportion of partial compliance. Figure 15B presents the chi-square statistics for the relationship between comorbidities and follow-up care compliance. The highest chi-square values are observed for cardiovascular disease (chi-square = 7.68, p-value = 0.021), stroke history (chi-square = 6.75, p-value = 0.035), and Parkinson’s disease (chi-square = 6.03, p-value = 0.049), indicating significant associations between these comorbidities and lower compliance with follow-up care in the study population (Figure 15).

## Discussion

The study on elderly patients with neck of femur (NOF) fractures emphasizes the critical role of comorbidities in shaping treatment outcomes, recovery trajectories, and long-term prognosis. The cohort of 163 patients, with a mean age of 80.23 ± 9.02 years, offers valuable insight into how chronic conditions influence recovery in this vulnerable population. Previous research corroborates the finding that the elderly often present with a high burden of comorbidities such as diabetes mellitus, hypertension, and cardiovascular diseases, which significantly alter the clinical course following fractures [17,18].

Comorbidities have emerged as key predictors of both mortality and prolonged functional disability after NOF fractures. The preoperative functional status, assessed through tools like the Barthel Index, is notably influenced by the presence of comorbid conditions, particularly cardiovascular disease and chronic pain disorders. These factors can exacerbate pre-existing disabilities and hinder rehabilitation efforts, creating a complex clinical environment that necessitates individualized management strategies. Research has shown that a high Charlson Comorbidity Index (CCI) correlates with poorer functional recovery at 1-year follow-up, especially in older adults undergoing hip fracture surgery [19,20,21].

The interaction between pain levels and comorbidities is particularly complex. Conditions such as anemia and diabetes mellitus may intensify acute pain responses, highlighting the need for comprehensive pain management approaches. In this cohort, 32.5% of patients reporting severe pain also had anemia, indicating the necessity for preoperative and postoperative strategies to manage this comorbidity and enhance pain relief efforts [21,22]. Furthermore, comorbidities like depression and chronic pain syndromes may lower pain thresholds, thereby complicating recovery [23,24].

Postoperative complications also appear to be influenced by the presence of comorbid conditions. Among the study population, 31.90% experienced complications such as hyperglycemia and volume overload, particularly in those with pre-existing conditions like diabetes and cardiovascular disease [25,26]. These findings highlight the importance of preoperative assessments to optimize patients' health statuses prior to surgery, reducing the risk of postoperative complications.

The presence of comorbidities also significantly affects hospital length of stay and the timing of surgery. Patients with a history of stroke or chronic kidney disease were more likely to experience extended hospitalizations, often due to delays in surgical intervention for stabilization [27,28]. These prolonged stays not only extend recovery times but also increase the likelihood of additional complications, underscoring the need for streamlined surgical processes to address the challenges faced by patients with multiple medical conditions [29,18].

Rehabilitation after NOF fractures is also affected by the complexity of comorbidities. Our findings suggest that patients with anemia and cardiovascular diseases required longer durations of physical therapy, emphasizing the need for rehabilitation protocols tailored to the specific limitations imposed by these conditions [25,30]. Interestingly, the nature of the rehabilitation, whether inpatient or outpatient, was less influenced by comorbidities, suggesting that functional impairments may be a more significant determinant of therapeutic intensity than the comorbidities themselves [31].

The study also explored follow-up care compliance, revealing that cognitive and physical impairments associated with comorbidities impede patients' ability to adhere to follow-up protocols. With only 41.71% of patients fully complying with follow-up care, strategies to proactively engage these patients are essential for improving outcomes [32,33]. Conditions such as Parkinson’s disease and a history of stroke were associated with significantly lower compliance rates, underscoring the need for healthcare systems to adapt to the unique challenges faced by this patient group [22,34].

While the type of surgical intervention (e.g., open reduction and internal fixation (ORIF)) did not exhibit a strong correlation with the prevalence of comorbidities, it was evident that recovery outcomes varied based on the presence of pre-existing health conditions. Therefore, although the choice of surgical method may not be directly influenced by comorbidities, the post-surgical care plan must effectively integrate these considerations to optimize patient recovery [18,19,35].

The clinical implications of these findings suggest that a patient-centric approach to care is essential. Managing comorbidities prior to surgery is critical for improving postoperative outcomes. Targeted interventions to stabilize conditions such as diabetes and cardiovascular diseases before surgery may lead to better recovery trajectories [31,36]. Additionally, rehabilitation strategies should be individualized to account for the functional limitations caused by multiple comorbidities [37,21]. Finally, ongoing patient education is vital for improving adherence to follow-up care, especially for patients who face complex health challenges beyond their fracture [38,39].

This study has several limitations that must be considered when interpreting the results. The retrospective design limits the ability to establish causality, and selection bias may affect the generalizability, as the sample may not represent all elderly patients with NOF fractures. The reliance on hospital records introduces the possibility of incomplete or inaccurate data, particularly in documenting comorbidities and postoperative complications. Additionally, the study's single-center design reduces external validity, and unmeasured confounders, such as psychological factors and patient adherence to rehabilitation, were not fully addressed. The absence of a control group further restricts direct comparisons between patients with and without comorbidities. Moreover, the study lacked long-term follow-up data, preventing the examination of the enduring effects of comorbidities on recovery. Variations in the severity of comorbidities were not considered, nor was the impact of specific treatments for these conditions assessed, potentially underestimating the role of medical management in recovery. These factors highlight the need for prospective, multicenter studies with control groups and long-term follow-up to validate and expand upon these findings.

## Conclusions

In conclusion, our study highlights the critical role that comorbidities play in the recovery of elderly patients with neck of femur fractures. Anemia, cardiovascular diseases, diabetes, hypertension, and stroke history were strongly associated with poorer recovery outcomes, including prolonged hospital stays, increased postoperative complications, and longer rehabilitation durations. These findings underscore the need for comprehensive preoperative assessment, tailored treatment plans, and effective postoperative management to improve the recovery and quality of life for elderly patients with NOF fractures. Addressing these comorbidities in a holistic and individualized manner is essential for optimizing recovery and minimizing complications in this high-risk patient group.
